# Immune responses triggered by HIV/AIDS vaccine candidates, derived from MVA-B, with deletions in several immune regulatory genes

**DOI:** 10.1186/1742-4690-9-S2-P302

**Published:** 2012-09-13

**Authors:** J García-Arriaza, P Arnáez, CE Gómez, M Esteban

**Affiliations:** 1 Centro Nacional de Biotecnología. CSIC. Madrid, Madrid, Spain

## Background

Poxvirus vector Modified Vaccinia Virus Ankara (MVA) expressing HIV-1 Env, Gag, Pol and Nef antigens from clade B (termed MVA-B) is a promising HIV/AIDS vaccine candidate, as it was shown in the results obtained from a phase I clinical trial.

## Methods

To try to improve the immunogenicity elicited by MVA-B we have generated and characterized the innate immune sensing and the in vivo immunogenicity profile of new optimizing MVA-B vaccine candidates, which contains deletions in one, two or three different immunomodulatory vaccinia virus (VACV) genes blocking the same signaling pathway, involved in the induction of type I IFN.

## Results

The innate immune signals elicited by these MVA-B deletion mutants in human macrophages showed an up-regulation of the expression of IFN-β and IFN-α/β-inducible genes. A DNA prime/MVA boost immunization protocol in mice revealed that these MVA-B deletion mutants were able to induce strong and polyfunctional HIV-1-specific CD4+ and CD8+ T-cell adaptive and memory immune responses, which were mostly mediated by CD8+ T cells with an effector phenotype. CD4+ T-cell responses were mainly directed against Env in MVA-B and all the MVA-B deletion mutants. However and interestingly, while MVA-B induced preferentially Env- and Gag-specific CD8+ T-cell responses, MVA-B deletion mutants induced more GPN-specific CD8+ T-cell responses. Moreover, an enhanced HIV-1-specific lymphoproliferative response was observed with the MVA-B deletion mutants. Furthermore, MVA-B and MVA-B deletion mutants were also able to induce antibodies against Env.

## Conclusion

These findings revealed that deletion in MVA-B of VACV genes that act blocking the same signaling pathway confers an immunological benefit by inducing innate immune responses and increasing the magnitude, quality and durability of the HIV-1-specific T-cell immune responses. Our observations focused the use of highly optimizing MVA-based vectors as more potent HIV-1 vaccines.

